# Draft Genome Sequence of Rice Orange Leaf Phytoplasma from Guangdong, China

**DOI:** 10.1128/genomeA.00430-17

**Published:** 2017-06-01

**Authors:** Yingzhi Zhu, Yuange He, Zheng Zheng, Jianchi Chen, Zhiyi Wang, Guohui Zhou

**Affiliations:** a Guangdong Province Key Laboratory of Microbial Signals and Disease Control, College of Agriculture, South China Agricultural University, Guangzhou, Guangdong, China; b USDA-ARS, San Joaquín Valley Agricultural Sciences Center, Parlier, California, USA

## Abstract

The genome of rice orange leaf phytoplasma strain LD1 from Luoding City, Guangdong, China, was sequenced. The draft LD1 genome is 599,264 bp, with a G+C content of 28.2%, 647 predicted open reading frames, and 33 RNA genes.

## GENOME ANNOUNCEMENT

Rice orange leaf disease was first discovered in Thailand in 1960 (1) and later in other parts of southeastern and eastern Asia (2, 3). In China, the disease was first reported in Yunnan Province in 1978 (4) and then spread to other rice-producing areas in southern China (5). The disease is associated with an unculturable rice orange leaf phytoplasma (ROLP) belonging to the “*Candidatus* Phytoplasma asteris” (16 SrI) group (2) that comprises numerous related phytoplasmas, including the onion yellow phytoplasma, which has its genome completely sequenced (6). The inability to culture ROLP presents a major obstacle for the bacterial study and therefore disease control. With the development of next-generation sequencing (NGS) technology, whole-genome sequencing of phytoplasmas is becoming feasible for many laboratories for this pathogen study. Genomic analysis has become an efficient and effective approach to generate a significantly large amount of genetic information for biological characterization of unculturable bacteria. In the present study, we report the draft genome sequence of ROLP from Guangdong Province in China.

Rice leaves with typical orange yellowing symptoms were collected from Luoding City, Guangdong Province, China, in 2016. The sample was designated ROLP-LD1. A modified cetyltrimethylammonium bromide (CTAB) method was used for DNA extraction (7). Extracted DNA was quantified by using a NanoPhotometer Pearl 61010-1 instrument (Implen GmbH, Munich, Germany). DNA sequencing was conducted using the Illumina HiSeq 2000 platform (Illumina, Inc., San Diego, CA).

A total of 42,886,932 reads (~12 Gb) with a mean of 150 bp per read were generated from the ROLP-LD1 sample. *De novo* assembly was performed using CLC Genomics Workbench 7.5. A total of 16 contigs were identified as ROLP-LD1 sequence by using onion yellow phytoplasma genome sequence (accession number NC_005303) as a reference through a BLASTn search, with E value setting at 10^-5^ and word size setting at 28 bp (8). Efforts were made to fill gaps between contigs by designing primers, PCR, and amplicon sequencing with Sanger’s method. The final assembly contained a total of 8 contigs ranging from 5,727 bp to 197,634 bp, with ~400× coverage. The draft genome sequence of ROLP-LD1 comprised 599,264 bp, with a G+C content of 28.2%. Sequence annotation was performed by the RAST server (http://rast.nmpdr.org) (9). The ROLP-LD1 genome was predicted to have 647 protein-coding genes and 33 RNA genes. Based on the 16S rRNA gene sequence, the ROLP-LD1 was confirmed to be a member of the “*Candidatus* Phytoplasma asteris” (16 SrI) group, with 99% similarity to the published ROLP 16S rRNA gene sequence JX290547 from India (2).

### Accession number(s).

This whole-genome shotgun project has been deposited at DDBJ/ENA/GenBank under the accession no. MIEP00000000 for the ROLP chromosome. The version described in this paper is version MIEP00000000.
